# Heterogeneity Analysis of the Effects of Haze Pollution on the Health of Left-Behind Children in Urban and Rural Areas in China

**DOI:** 10.3390/ijerph182111596

**Published:** 2021-11-04

**Authors:** Juan Wang, Qiuhe Wei, Qing Wan, Hai Li

**Affiliations:** 1 School of Management, Wuhan Institute of Technology, Wuhan 430205, China; wangjuan@wit.edu.cn (J.W.); qh06170318@163.com (Q.W.); Qingwan@wit.edu.cn (Q.W.); 2 School of Business Administration, Zhongnan University of Economics and Law, Wuhan 430073, China

**Keywords:** left-behind children, children’s health, raster PM2.5, urban and rural areas differences, heterogeneous effects

## Abstract

Differences in economic development, public services, production, and lifestyle between urban and rural areas lead to significant differences in people’s attitudes and abilities to cope with environmental pollution. Furthermore, environmental pollution has heterogeneous effects on the health of individuals in urban and rural areas. The article takes the health of left-behind children as an entry point to analyze the impact of haze pollution on the health of urban and rural left-behind children. Using children’s survey data from the China Health and Nutrition Survey and the urban and rural raster PM2.5 data from 2000 to 2015, this study applies a logit model to analyze the heterogeneity of the impact of haze pollution on the health of left-behind children. This research finds that, first, the health effects of haze pollution on rural left-behind children are more severe than those on rural children not left behind. Moreover, the same results are not present in the sample of urban left-behind children. Second, the health of left-behind children is more vulnerable to haze pollution than the others when neither parent is at home in rural areas. Third, no evidence proves that haze pollution has more severe health effects on rural children aged 0–6 years with parents away from home. Meanwhile, haze pollution will more easily influence the health status of left-behind children aged 7 years and above in rural areas due to their parents’ absence. Fourth, the finding that haze pollution significantly affects the health of left-behind children with parents away from home only applies to the central and western rural samples in China.

## 1. Introduction

The increase in the number of hazy days poses a great threat to human health [1]. The Human Development Report 2019 published by the United Nations Development Program states that environmental pollution and environmental disasters tend to hit vulnerable groups more severely and are relatively more harmful. Furthermore, environmental inequality reflects the unequal spatial distribution of environmental risks, hazards, environmental public services, and so on [2]. The shift of urban industries to remote rural areas has caused an increase in the concentration of haze in rural areas. However, the investment in public facilities for environmental protection has not increased simultaneously. As a result, the health problems of rural residents have become increasingly prominent, and rural residents have become the largest victims of environmental inequality. In particular, children are more sensitive to polluted air due to their physique; hence, most of the literature has paid extensive attention to the health hazards of environmental pollution on children [3,4]. However, the relevant analysis of left-behind children remains insufficient. The number of left-behind children, a social phenomenon specific to China’s dualistic economic structure, has risen as a result of labor mobility. The act of one or both parents being forced to leave their children behind, such as in their registered household, has a range of effects on children’s health [5]. However, the literature analyzing haze pollution and health has insufficiently focused on left-behind children, a high-risk group for environmental stress, as the subject of study.

As a special group of children, left-behind children suffer more serious health hazards in the face of haze pollution due to the lack of corresponding coping measures, coping abilities, and so on. On the one hand, the huge difference in economic development and public services has led to several dissimilarities in attitude and ability to cope with environmental pollution between urban and rural areas. In addition, numerous polluting enterprises have moved to remote rural areas with lax environmental control and large environmental capacity due to increasingly strict environmental regulations in cities. Consequently, this movement has led to increasingly serious local haze pollution. Children’s imperfect development of their immune system and respiratory system makes them a high-risk group for haze pollution stress, thus causing them to suffer from more significant health effects [6]. In cities with relatively better public services and environmental awareness, parents cope with the negative effects of environmental pollution on their children’s health by paying for preventive equipment or being absent from work [7]. In contrast, left-behind children in rural areas lack proper care and protection, especially effective interventions to cope with haze pollution. The absence of their parents engender children’s exposure to the haze environment.

On the other hand, the ecological environment in numerous rural areas is relatively fragile. People’s lives depend more on the natural environment even if the ecosystem has been polluted. The vast majority of left-behind children are raised by intergenerational elders. Given that these guardians are generally older, the left-behind rural children are more or less involved in household and agricultural work, thus having more exposure to haze pollution. Unlike those in urban areas, most guardians in rural areas do not educate their left-behind children about environmental pollution and other issues, thus lacking effective intervention between left-behind children and environmental pollution. As a result, left-behind children have no awareness or measures to deal with the damage of haze pollution on their health. Both the difference in the production practices of burning straw to increase soil fertility and the difference in lifestyles involving burning non-clean energy for cooking and heating lead to the possibility that the health effects of haze may differ between urban and rural left-behind children.

The publicity and education on environmental pollution countermeasures are far from enough. As children are the main players in future social development, determining whether haze pollution has a severely negative effect on the health of left-behind children and identifying the mechanism of the impact are critical areas of study for China’s strategy to revitalize the countryside. The present article uses data from the China Health and Nutrition Survey (CHNS) and urban–rural raster PM2.5 concentration data (the index measures haze pollution levels) to analyze the impact and heterogeneity of haze pollution on the health of left-behind children using a logit model. Compared with previous studies, this work uses relatively detailed and valid data to perform an empirical test on the relationship between environmental pollution and the health of left-behind children in urban and rural areas. The investigation of haze pollution and health from the perspective of left-behind children not only helps to enrich the research on health economics but also to provide empirical evidence for improving the health of left-behind children in rural China.

## 2. Research Overview

Left-behind children are commonly identified as children under the age of 18 with both or one parent absent from home. Qu et al. (2019) analyzed from the perspective of the mental health of left-behind children has concluded that the presence or absence of parents living near their children brings about great changes in children’s psychological feelings; moreover, studies have determined that anxiety, capriciousness, introversion, and loneliness are common psychological problems left-behind children face [8]. Furthermore, Liu et al. (2019) studied from an economic perspective has generally concluded that the state of being left behind is detrimental to children’s health, especially in the absence of the mother [9]. Zhao et al. (2014) argued that left-behind children have poorer health than non-left-behind children from the perspective of nutritional development and disease [10]. However, the literature using BMI as a measure of children’s health to analyze the impact of stay-at-home arrangements has not reached uniform conclusions. Some studies have found a significant increase in the BMI in left-behind children [11]. Meanwhile, other works have found that the BMI of left-behind children whose fathers are away while their mothers are at home remains unaffected. The selection of control variables in the factors influencing the health of left-behind children and the definition of the dependent variable of children’s health have evolved and improved in the research. In addition, the use of samples has evolved from simple research to detailed, large-scale analyses in multiple provinces.

As environmental problems continue to worsen, the number of studies on air pollution and health is soaring. Air pollution has been well established to have adverse health effects [12]. Much of the literature focuses on infants and young children, which partially reduces the endogenous error because of their health characteristics. Seema (2009) analyzed the relationship between pollution concentrations and infant and child mortality through a quasi-experiment of increased air pollution from forest fires; the author found that air pollution from fires resulted in significantly lower infant and child survival and that disparity was more severe in poorer areas [13]. A quasi-experiment with reduced air SO_2_ concentrations due to an oil refinery strike found a significant increase in the weight and gestational age of local newborns. Cesur et al. (2015) analyzed changes in infant mortality using data on reduced provincial air pollution due to the expansion of natural gas facilities in Turkey; they found that the use of natural gas significantly reduced infant mortality [14]. Meanwhile, a study using German air monitoring data analyzed in combination with survey data found that CO pollution reduced fetal body weight and had a significant effect on neurological disorders in infants [15]. Moreover, an analysis using CO pollution data from Mexico revealed that increased CO pollution reduced infant and child survival [16]. Sunyer et al. (2017) followed 2687 elementary school students in 39 schools in Barcelona and found that traffic-related air pollution was potentially harmful to the neurodevelopment of elementary school students, thus directly affecting their attention. Meanwhile, He et al. (2016) used exogenous changes in air quality during the 2008 Olympic Games in Beijing, China to estimate the effect of air pollution on mortality; they found that the effect of air pollution was the same across genders but had the greatest effect on children under the age of 10 years [1].

Throughout the above literature, studies have analyzed the health of left-behind children in terms of intrinsic and extrinsic factors. Moreover, the literature on health economics has argued for the health hazards of air pollution on children from different pollution perspectives. However, the existing literature is less concerned with whether left-behind children, who emerge in the special development context of China, face more serious environmental and health problems. Analyzing the issue of urban and rural left-behind children and haze pollution with scientific and valid data is a useful addition to the study of children’s health.

## 3. Influence Mechanisms and Assumptions

### 3.1. Impact of Environmental Regulations and Public Service Differences between Urban and Rural Areas

The environmental regulations and policies set by the central government itself are not differentiated between urban and rural areas, but China has a governmental governance system that combines administrative centralization and economic decentralization. Local governments have a great deal of decision-making power over local affairs. So environmental regulations are implemented differently in various regions due to dissimilarities in the degree of economic development, public infrastructure, and other conditions. These characteristics lead to large differences in individuals’ attitudes and abilities to deal with environmental pollution between regions. With the dramatic improvement of people’s living standards, environmental protection has received widespread attention, and various environmental control policies, regulations, and measures have emerged. However, in some remote areas, highly polluting and energy-intensive industrial development is still the mainstay of the local economy. To cope with the demand of environmental protection, numerous polluting enterprises upgrade their technology. However, in the face of the increasingly strict environmental regulations in cities, some polluting enterprises opt to relocate to remote areas, such as suburbs and rural areas, where the economy is relatively backward, environmental control is relatively lax, and environmental capacity is larger [17]. Furthermore, local governments, for the sake of economic development, have introduced relevant preferential strategies to attract capital to enter and establish various industrial parks in suburban and rural areas. These developments have intensified the transfer of haze pollution between urban and rural areas.

Importantly, when companies and local governments select sites for industries that may cause haze pollution, most of these entities avoid residential areas with strong resistance and opt for remote areas, such as rural areas, with fewer social resources, lower political and social influence, and lower economic and educational capacity [18]. As a result, environmental inequality increases. The consequence of the multi-cause formation is that some rural areas have to face increasingly serious haze pollution. Unfortunately, the entry of polluting companies has not ushered in a massive increase in public services in remote rural areas, especially environmental protection. As a result, these areas are likely to suffer more health losses even when faced with the same hazy environment as urban areas.

Compared with adults, children are a high-risk group for environmental stress. In addition, some of their organ systems, including the immune system and the respiratory system, are still in the developmental stage. Hence, children are more sensitive to haze pollution and suffer more significant and serious health injuries. As a special group among them, most rural left-behind children are taken care of by their grandparents and other intergenerational relatives because their parents need to go out to work to improve their family situation. Parental intervention in children’s education can prevent harm from pollution [19]. Compared with urban parents with better education and knowledge of environmental protection, older rural guardians do not have the relevant knowledge base on how to carry out haze pollution prevention and protection. Thus, they are unlikely to educate their left-behind children intergenerationally. Unlike some parents in cities who cope with poor air quality by paying for defensive equipment or through absenteeism, rural left-behind children generally lack effective interventions for haze pollution. Thus, the latter suffers from more health losses than the former. As such, Hypothesis 1 is proposed:

**Hypothesis** **1.** *The health effects of haze pollution on left-behind children in rural areas are more significant than those on left-behind children in urban areas.*

### 3.2. Impact of Differences in Production and Lifestyle between Urban and Rural Areas

The natural, climatic, and other environments in cities are more suitable for human habitation and have more stable ecosystems. In contrast, rural areas, especially remote areas, have relatively fragile ecological environments, and people rely more on the natural environment for survival. Living at the mercy of the elements, coupled with the decrease in the number of laborers brought about by migrant workers, drives the families left behind to increase their income or agricultural production, which is mostly achieved by straw burning, excessive use of pesticides, and other ways of production. While these activities increase agricultural production, they more often destroy the ecological balance and increase the possibility of haze pollution. Left-behind children in rural areas are also forced to participate in agricultural labor due to the absence of their parents and the aging of their relatives in the next generation. Therefore, the increased chance of direct exposure to the haze environment makes them more vulnerable to negative health effects.

The difference in lifestyles between urban and rural areas is also one of the reasons why haze pollution more severely affects the health of left-behind children in rural areas. Remote rural areas mostly use non-clean energy for daily life, such as loose coal and straw, which are one of the major sources of haze pollution [20]. The burning of straw sharply increases the concentration of soot, particulate matter, and other pollutants in the air. The burning of poor-quality coal in cooking and heating sharply increases the concentration of particulate matter, sulfur dioxide, carbon monoxide, and other pollutants in the air, all of which can seriously damage health. The widespread use of clean energy sources, such as natural gas, in cities reduces haze pollution from the improper use of domestic energy sources. However, rural children are directly or indirectly exposed to haze pollution from the burning of non-clean energy sources.

Traditional production and lifestyle, combined with the lack of effective interventions between pollution and health, put rural left-behind children at risk of haze pollution without appropriate coping measures and coping abilities. However, this effect varies across age groups. Children in the younger age group (aged 0–6 years old) are less likely to encounter haze pollution because of their inability to undertake household or productive work and their limited range of activities. Meanwhile, children aged 7 years and older are more severely exposed to the health risks of haze pollution because they are old enough to undertake agricultural work. In conclusion, left-behind children aged 7 and above are exposed to more serious health hazards from haze pollution. Thus, Hypothesis 2 is proposed:

**Hypothesis** **2.** *The health effects of haze pollution on rural left-behind children aged 7 years and above are more significant than those on rural left-behind children aged 0–6 years*.

## 4. Model Settings and Data Sources

### 4.1. Model Settings

Given the definition of child health status data and the purpose of the study, the article uses a logit model to analyze the effect of environmental pollution on the health of left-behind children. The empirical model is set up as follows:

(1) $$health_{i,t}=\;\propto +\beta_{1}none\times PM2.5_{i,t}+rstewater\;\beta_{2}nomo\times PM2.5_{i,t}+\beta_{3}nofa\times PM2.5_{i,t}+\beta_{4}PM2.5_{i,t}\;+none_{i,t}+nomo_{i,t}+nofa_{i,t}+\theta X_{i,t}+\eta \delta_{i}+\gamma \varphi_{t}+\mu_{i,t},\cdots \cdots \cdot \cdot$$

where i is the child and t is the time. health is the child’s physical health status, which is measured using the indicator “sick or chronically or acutely ill in the past four weeks ……”, with health taking a value of 1 when these conditions are present and 0 otherwise. Given the availability of data on children’s health status and database limitations, most of the literature uses indicators such as self-rated health and illness to measure children’s health status over time. However, most of the data on children’s self-rated health are missing; thus, we use the sickness indicator to measure health, which is a more accurate indicator of children’s health and is a valid indicator of their health status [5]. In addition, the article measures the absence of parents of left-behind children. The categories both parents not at home (none), mother only not at home (nomo), and father only not at home (nofa) are all measured by 0 to 1 dummy variables. The interaction terms between the three types of dummy variables and pollution indicators are measured by the following: none×PM2.5, nomo×PM2.5, and nofa×PM2.5. PM2.5 is an indicator of haze pollution. In addition, raster PM2.5 data are used to measure the degree of haze pollution between urban and rural areas roughly.

X denotes a series of control variables. The basic control variable is basic personal information, while the remaining control variables are family situation and parental education. Basic personal information is commonly used as a control variable for health economics, including age, height, weight, education, and health insurance status [21]. Age is a proxy variable for the health depreciation rate and is measured by the actual age of the individual (age). Height (height) and weight (weight) are both expressed as actual individual values. Education is measured by the number of years of formal education received in a formal school. Increased education means increased access to health information and pollution protection information. Having health insurance (insurance) is a dummy variable. In addition, having health insurance means that when an individual’s health is impaired, his or her access to this service increases because of the relatively low price of medical services. At the same time, most hospitals that cooperate with health insurance can provide reasonable treatment options, which can contribute to the improvement of an individual’s health [22].

Household situation includes the number of people in the household (house size), household income, and household sanitation. Household income is measured by the deflated annual per capita household income (income). High income means that individuals have more opportunities to purchase healthy services, food, better medical care, quality living environment, and so on, which are beneficial to improve health [23]. Quality sanitation is an effective guarantee of health. As such, household sanitation is measured by two dummy variables: Whether the household drinking water is tap water (tap water) and whether the household has a working flush toilet (water closet).

Father’s education (education_f) and mother’s education (education_m) are measured by the number of years of formal education that parents received in a formal school. Fathers and mothers with higher education are more likely to have more knowledge about children’s health and to have some awareness of the relationship between environmental pollution and children’s health. $\varphi_{t}$ denotes year fixed effects, $\delta_{i}$ denotes individual fixed effects, and $\mu_{i,t}$ is the error term. Given that health indicators are 0–1 variables, this study uses a logit model for analysis.

### 4.2. Data Sources

The article uses panel data from the CHNS, a long-term follow-up study conducted jointly by the Institute of Nutrition and Food Safety of the Chinese Center for Disease Control and Prevention and the Population Center of the University of North Carolina in the United States. The survey began in 1989 and has been conducted 10 times through 2019. It has a multistage stratified random sample of approximately 4400 households and 19,000 individuals in urban and rural areas in the provinces of Liaoning, Heilongjiang, Shandong, Jiangsu, Henan, Hubei, Hunan, Guizhou, and Guangxi in China. The CHNS database contains indicators on demographic characteristics, economy, public resources, and health. Since 1991, the research data have relatively complete indicators related to child retention and children’s health. Combined with PM2.5 raster data, the article uses research data from 2000 to 2015.

PM2.5 concentration data are obtained from the Global PM2.5 Annual Average Concentration Raster Dataset (V4.GL.03) constructed by the Atmospheric Composition Analysis Group (ACAG) at Dalhousie University. The information can be downloaded from http://fizz.phys.dal.ca/~atmos/martin/?page_id=140, from 16 March 2019 to 5 May 2020. To obtain the average concentration data at the county and city levels, the administrative district boundary vector data are used as a mask. The spatially corrected raster data are cropped and partitioned for statistics using ArcGIS software. The administrative boundary vector data are obtained from the 1:100 China Basic Geographic Information Database provided by the National Basic Geographic Information Center.

The article divided the statistical description of left-behind children into rural and urban samples, and the description of each statistical variable is shown in Table 1 (Less than 10 of the matched data using key variables had one or both parents dead, for the accuracy of the findings, the data did not include data on the death of both or either parent.). Health status is a 0–1 variable. Furthermore, the mean value of the rural sample is slightly higher than the health status of the urban sample, thus indicating that the health status of children in the rural sample is lower than that of the urban sample. However, the PM2.5 value of the urban sample is higher than that of the rural sample. The percentage of left-behind children in the rural sample whose parents are both absent from home is 2.9%, and the mean value of left-behind children’s health is 0.102. Meanwhile, the percentage of left-behind children whose mothers are absent from home only is 2.1%, and the mean value of left-behind children’s health is 0.080. The percentage of left-behind children whose fathers are absent from home only is 13.8%, and the mean value of left-behind children’s health is 0.032. In the urban sample, 2.9% of left-behind children have both parents not at home, and the mean value of left-behind children’s health is 0.038. In addition, 5.0% of left-behind children only have their mothers not at home and the mean value of left-behind children’s health is 0.018. 4.3% of left-behind children only have their fathers not at home, and the mean value of left-behind children’s health is 0.015. The mean values of the rural and urban samples for the gender variable have no significant difference, thus indicating that the ratio of males to females is balanced in both samples.

## 5. Empirical Findings

### 5.1. Haze Pollution and the Health of Left-Behind Children

Table 2 shows the basic regression results of the health of left-behind children and PM2.5 pollution indicators. All regressions in Table 2 consider individual effects and year effects. Columns (1)–(3) of Table 2 are the regression results for left-behind children whose sample is living in rural areas. Then, columns (4)–(5) of Table 2 are the regression results for left-behind children whose sample is living in urban areas. The last column is the regression results for the full sample. Column (1) of Table 2 does not include control variables. Column (2) controls for the children’s age, height, and weight. Meanwhile, column (3) continues to include the variables of children’s education level and whether they have health insurance. The regression results of the three logit models reveal that the coefficient of the PM2.5 pollution indicator in rural areas is significantly positive at the 10% significance level, which indicates that PM2.5 pollution is detrimental to the health of rural children. The estimated coefficients of the interaction term between rural left-behind children and pollution indicators show that the estimated coefficients of the interaction term between the pollution indicators and the dummy variable of left-behind children with neither parent at home are significantly positive at the 5% level of significance. Meanwhile, the rest of the interaction terms are not significant. The results indicate that PM2.5 pollution is more detrimental to the health of left-behind children whose parents are not at home than to the health of other children in rural areas. The above analysis demonstrates that rural left-behind children whose parents are not at home are more likely to suffer from health impairment due to PM2.5 pollution than other children in rural areas.

The regression results in columns (4)–(6) of Table 2 reveal that the health effects of the pollution indicators are significantly adverse regardless of the adjustment of the control variables. However, none of the interaction terms between the pollution indicators and the indicators of urban children’s retention are significant. This outcome implies that no evidence has proven that urban left-behind children suffer more health losses due to PM2.5 pollution hazards than other children. Compared with rural areas, large cities have more advanced and complete public facilities, such as medical services and environmental protection, because of their dense population and other reasons; thus, these facilities improve individual health status [24]. The results of the total sample test in the last column of Table 2 suggest the absence of evidence on whether the health status of left-behind children is more vulnerable to PM2.5 pollution than that of other children.

The combined regression results in Table 2 reveal that PM2.5 pollution affects the health of both rural and urban children. Meanwhile, the coefficients indicate that rural areas are less affected than urban areas. However, when analyzing its effect on the health of left-behind children, PM2.5 pollution has been found to have a significant effect on the health of left-behind children in rural areas relative to other children. In addition, the conclusion does not hold in the urban sample. The health of rural left-behind children is more at risk from environmental pollution when both parents are not at home—a finding that supports Hypothesis 1.

Several reasons may account for this outcome. First, left-behind children living in cities may be less likely to suffer from health hazards due to environmental pollution because urban areas have more environmental management measures, better infrastructure, better education, and a more hygienic family environment. Second, left-behind children in rural areas are more likely to suffer from health hazards due to poor economic development, poor education, poor medical conditions, and lack of health awareness. Third, compared with intergenerational guardians, both parents are more attentive and concerned about their children’s health. Hence, the health of left-behind children whose parents are not at home is more vulnerable to environmental pollution.

### 5.2. Robustness Checks

Robustness tests are conducted mainly by the following methods: adding more control variables and removing the effects of municipalities. Considering the differences in development between regions in terms of economy, education, and so on, which have the potential to affect the robustness of the findings, this study includes regional fixed effects in each model. Considering the influence of factors such as family income, health, and parents’ educational background on children’s health, this article further controls for these influencing factors, and the regression results are shown in columns (1) and (3) of Table 3. The test results of the PM2.5 pollution index and the interaction term variables of dummy variables for left-behind children when both parents are not at home are also consistent with the basic findings in Table 3. Given the special political and economic background of the municipality, which may affect the regression results, the article removes the municipality for further robustness tests, as shown in columns (2) and (4) of Table 3. Both regressions include a series of control variables for household income and population, water use, and parental education. Moreover, the regression results reveal that the findings of the interaction term between pollution indicators and left-behind children confirm the robustness of the findings in Table 2.

## 6. Heterogeneity Impact Analysis

### 6.1. Age Heterogeneity

To expand the exploration on the effect of environmental pollution on the health of rural left-behind children, the article is divided into different samples for heterogeneity analysis. In addition, this research attempts a regression analysis of the sample of rural children by gender. The regression results do not differ possibly because both males and females are susceptible to air pollution. Therefore, the health of rural left-behind children as a result of environmental pollution does not differ according to gender. The article further conducts subsample regression analysis on rural children of different age groups. The regression results are shown in columns (1)–(3) of Table 4.

All regressions in Table 4 include all children’s individual and household control variables, controlling for individual and year effects. Among them, the regression results of the subsample of rural children aged 0–6 years in column (1) reveal that none of the interaction terms between pollution indicators and child retention are significant. This outcome indicates that the effect of PM2.5 pollution on the health of rural children aged 0–6 years left behind does not differ from that of children not left behind. With the gradual increase in concern for the growth and health of infants and young children in recent years in China, most of the rural infants and young left-behind children are well cared for. Compared with children of other age groups, children aged 0–6 years receive the most care, and the health of left-behind infants and toddlers is not significantly reduced by haze pollution compared with that of non-left-behind children.

Column (2) of Table 4 presents the regression results for the sample of rural children aged 7–12 years. The results reveal that the estimated coefficient of the interaction term is significantly positive. This outcome indicates that PM2.5 pollution has a more significant effect on the health of children aged 7–12 years who are left behind than on the health of non-left behind children aged 7–12 years. Most of the children at this age start to attend elementary school. Once they are in school, their elderly guardians hand over part of their care responsibilities to the school and relax their close protection of their children. Education in elementary school increases children’s awareness of the hazards of environmental pollution. However, a weak sense of self-control, neglect by guardians, and external environmental hazards are likely to increase children’s exposure to polluting emissions, which in turn affects their health.

Column (3) of Table 4 demonstrates the regression results for the subsample of rural children aged 13–18 years. The results reveal that the estimated coefficient of the interaction term is significantly positive. Thus, these findings indicate that the effect of PM2.5 pollution on children’s health relative to the rest of the children is also present in the sample of rural children aged 13–18 years whose parents are both absent from home. The combined regression results in Table 4 show that left-behind children aged 7–18 are more exposed to haze pollution hazards due to the absence of parental roles. The above empirical results verify Hypothesis 2.

### 6.2. Geographical Heterogeneity

Considering the different degrees of economic and social development in the eastern, central, and western regions of China, are the regression findings also geographically heterogeneous? The regression results are shown in columns (4)–(6) of Table 4. The regression results of health and pollution indicators for rural left-behind children in the eastern region in column (4) of Table 4 reveal that the estimated coefficients of the interaction term are not significant. Thus, the presence or absence of rural left-behind children in the eastern region does not affect the impact of environmental pollution on the health of rural children. Meanwhile, the regression results for the central and western samples in columns (5)–(6) of Table 4 reveal that the estimated coefficients of the interaction terms are both significantly positive. This outcome indicates that the health of left-behind children is more affected by haze pollution in rural areas of central and western regions compared with the health of other children.

The three regression results demonstrate that the health of rural children in the eastern region is less affected by environmental pollution than in the central and western regions. This difference is related to the developed economy, abundant educational resources, and more comprehensive social development in the eastern region. In contrast, most of the elders in rural families in the central and western regions are not well educated and have insufficient knowledge about environmental pollution and health. In particular, the elderly in rural areas do not have the ability to prevent and solve environmental pollution. Consequently, rural left-behind children become exposed to serious environmental pollution and are susceptible to a decline in their health status.

## 7. Conclusions

The dichotomous structure of China’s urban and rural areas has led to the movement of rural labor to cities. The hollowing out of rural areas has gradually widened the difference in economic development between rural and urban areas. Meanwhile, the transfer of highly polluting enterprises to rural areas has increased pressures on rural environmental management and the rural haze pollution is getting worse. To improve the economic situation of families, both or one of the parents in rural families opt to go out to work. However, the lack of parental care in the increasingly severe situation of rural haze pollution also has an impact on the health of left-behind children. The article uses data from the CHNS and raster PM2.5 data to analyze the effects of haze pollution on the health of left-behind children and their heterogeneity using a logit model. The findings of the study are as follows.

First, the health effects of haze pollution on urban and rural left-behind children are heterogeneous due to the differences in environmental regulations and public accommodation. Compared with the health of urban left-behind children, the health of rural left-behind children is more vulnerable to the health hazards of haze pollution.

Second, compared with other left-behind children in rural areas, the health of left-behind children is more at risk from haze pollution when both parents are not at home. Parents in the family may be more attentive to the care and protection of children and more concerned about children’s health than intergenerational guardians or other guardians.

Third, whether parents stay behind does not significantly affect the relationship between haze pollution and children’s health in the sample of rural children aged 0–6 years. However, the health conditions of children aged 7–12 and 13–18 years who are left behind are more affected by haze pollution due to their parents’ absence. Meanwhile, the relationship between rural children’s health and environmental pollution in the eastern region does not appear to be heterogeneous in terms of whether parents stay behind. However, left-behind children in central and western rural areas are more likely to experience haze pollution as a health hazard when their parents are absent.

At the micro level, the health of left-behind children impacts personal development and family economy. At the macro level, improving the health status of left-behind children is also significant for the long-term development of the countryside and the harmonious social development of China. Therefore, the health of left-behind children in rural areas needs to be taken seriously, especially for those whose parents are both working outside their hometown.

The study has the following limitations: first, a rough urban–rural division. The existing division is defined based on household registration, but as population mobility becomes more frequent, this phenomenon can affect the accuracy of analyzing the health differences between urban and rural populations due to environmental pollution. Second, the choice of pollution indicators. Rural water pollution and heavy metal pollution are also not negligible and may have a greater impact on health. However, there is a lack of indicators related to urban–rural pollution.

## Figures and Tables

**Table 1:** Descriptive statistics of major variables.

Variable Name	Rural			Urban		
	Observations	Mean	Standard Deviation (s)	Observations	Mean	Standard Deviation (s)
health	4576	0.060	0.234	2045	0.058	0.237
PM2.5	4576	45.575	16.464	2045	49.485	11.733
none	4576	0.115	0.192	2045	0.029	0.098
nomo	4576	0.021	0.083	2045	0.050	0.218
nofa	4576	0.138	0.210	2045	0.043	0.118
gender	4576	0.513	0.500	2045	0.432	0.495
age	4576	12.510	4.315	2045	11.651	4.072
height(cm)	4576	146.677	20.339	2045	146.369	18.991
weigh(kg)	4576	39.958	14.235	2045	41.166	15.073
insurance	4576	0.619	0.486	2045	0.647	0.478
education	4576	17.673	5.523	2045	18.032	6.151
Income (Yuan)	4576	10,581.980	16,938.650	2045	12,527.850	37,228.080
house size (population number)	4576	4.933	1.431	2045	4.841	1.288
tap water	4576	2.229	1.055	2045	1.342	0.626
water closet	4576	3.556	2.051	2045	1.921	1.430

**Table 2:** Basic regression results of haze pollution and the health of left-behind children.

Variables	(1)	(2)	(3)	(4)	(5)	(6)	(7)
	Rural (Logit Model)			Urban(Logit Model)			All (Logit Model)
none × PM2.5	0.108 **	0.115 **	0.083 **	0.004	0.023	0.018	0.082
	(0.048)	(0.037)	(0.026)	(0.960)	(0.765)	(0.822)	(0.140)
nomo × PM2.5	0.057	0.062	0.050	0.020	0.032	0.007	0.041
	(0.183)	(0.163)	(0.245)	(1.000)	(1.000)	(1.000)	(0.368)
nofa × PM2.5	−0.074	−0.071	−0.040	−0.139	−0.146	−0.168	0.068
	(0.106)	(0.174)	(0.244)	(0.201)	(0.181)	(0.143)	(0.211)
PM2.5	0.009 *	0.012 *	0.010 *	0.065 ***	0.066 ***	−0.074 ***	0.037 ***
	(0.079)	(0.083)	(0.088)	(0.006)	(0.006)	(0.003)	(0.001)
none	−5.447 **	−5.715 **	−3.669 **	−0.768	−1.876	−1.631	−4.217 *
	(0.023)	(0.018)	(0.029)	(0.826)	(0.621)	(0.677)	(0.082)
nomother	−3.084	−3.119	−2.422	−3.526	−2.760	−4.736	−2.193
	(0.124)	(0.132)	(0.231)	(1.000)	(1.000)	(0.999)	(0.312)
nofather	2.968	2.722	1.638	6.219	6.665	7.839	2.574
	(0.109)	(0.186)	(0.350)	(0.244)	(0.217)	(0.168)	(0.226)
age		−0.079 *	−0.069 *		−0.084	−0.102	−0.073
		(0.082)	(0.061)		(0.224)	(0.157)	(0.109)
height		−0.010	−0.013 *		−0.015	−0.013	−0.009
		(0.338)	(0.093)		(0.218)	(0.311)	(0.401)
weight		0.014	0.010		0.004	0.005	0.013
		(0.132)	(0.176)		(0.733)	(0.683)	(0.191)
insurance			0.390 **			−0.011	0.405 *
			(0.012)			(0.964)	(0.061)
education			0.048 **			0.086 **	0.033
			(0.012)			(0.017)	(0.182)
Individual	Yes	Yes	Yes	Yes	Yes	Yes	Yes
Year	Yes	Yes	Yes	Yes	Yes	Yes	Yes
Observations	4576	4576	4576	2045	2045	2045	6580
R−squared	0.638	0.646	0.655	0.596	0.606	0.615	0.605

Note: ***, **, and * indicate the significance levels of 1%, 5%, and 10%, respectively. *p*-value in parentheses.

**Table 3:** Robustness test results.

Variables	(1)	(2)	(3)	(4)
	Rural (Logit Model)		Urban (Logit Model)	
	Add Control Variables	Remove the Effect of Municipalities	Add Control Variables	Remove the Effect of Municipalities
none × PM2.5	0.078 *	0.077 *	0.022	0.055
	(0.070)	(0.071)	(0.793)	(1.000)
nomo × PM2.5	0.029	0.029	0.046	0.056
	(0.532)	(0.532)	(1.000)	(1.000)
nofa × PM2.5	−0.063	−0.063	−0.178	−0.178
	(0.195)	(0.195)	(0.127)	(0.157)
income	−0.385 *	−0.383 *	−0.074	−0.011
	(0.078)	(0.078)	(0.772)	(0.970)
house size	0.044 *	0.044 *	0.089 **	0.095 **
	(0.083)	(0.083)	(0.013)	(0.019)
tap water	−0.000	−0.000	−0.000 **	−0.000
	(0.150)	(0.150)	(0.033)	(0.102)
water closet	−0.046	−0.046	−0.158	−0.165
	(0.352)	(0.352)	(0.147)	(0.163)
education_f	−0.155 *	−0.155 *	0.020	−0.049
	(0.069)	(0.069)	(0.936)	(0.859)
education_m	−0.130 ***	−0.130 ***	−0.001	0.027
	(0.006)	(0.006)	(0.996)	(0.803)
Control variables	Yes	Yes	Yes	Yes
Individual	Yes	Yes	Yes	Yes
Area	Yes	Yes	Yes	Yes
Year	Yes	Yes	Yes	Yes
Observations	4569	4562	2040	1921
R-squared	0.660	0.660	0.618	0.645

Note: ***, **, and * indicate the significance levels of 1%, 5%, and 10%, respectively. *p*-value in parentheses. Control variables included age, height, weight, child education, possession of health insurance, household income per capita, and household size variables.

**Table 4:** Heterogeneity regression results for rural samples.

Variables	(1)	(2)	(3)	(4)	(5)	(6)
	Age (Logit Model)			Region (Logit Model)		
	0–6 Years	7–12 Years	13–18 Years	Eastern Region	Central Region	Western Region
none × PM2.5	−0.093	0.130 *	0.210 *	0.084	0.092 *	0.073 **
	(1.000)	(0.073)	(0.052)	(0.887)	(0.092)	(0.170)
#omo × PM2.5	−0.097	0.026	0.020	0.221	0.157	0.081
	(0.500)	(0.418)	(0.499)	(1.000)	(0.974)	(0.431)
nofa × PM2.5	−0.149	−0.076	−0.025	−0.074	0.014	−0.116
	(0.999)	(0.401)	(1.000)	(1.000)	(1.000)	(0.515)
Control variables	Yes	Yes	Yes	Yes	Yes	Yes
Individual	Yes	Yes	Yes	Yes	Yes	Yes
Year	Yes	Yes	Yes	Yes	Yes	Yes
Observations	555	1536	2485	734	2772	1070
R-squared	0.760	0.759	0.792	0.635	0.775	0.580

Note: ** and * indicate the significance levels of 5%, and 10%, respectively. *p*-value in parentheses. Control variables included age, height, weight, child education, possession of health insurance, household income per capita, and household size variables.

## Data Availability

The data can be downloaded at: https://www.cpc.unc.edu/projects/china/data/datasets/index.html (accessed on 27 August 2021).
